# Encephalopathy in a Large Cohort of British Cerebral Autosomal Dominant Arteriopathy With Subcortical Infarcts and Leukoencephalopathy Patients

**DOI:** 10.1161/STROKEAHA.118.023661

**Published:** 2019-01-14

**Authors:** Anna M. Drazyk, Rhea Y.Y. Tan, Jonathan Tay, Matthew Traylor, Tilak Das, Hugh S. Markus

**Affiliations:** 1 From the Stroke Research Group, Department of Clinical Neurosciences, University of Cambridge, United Kingdom (A.M.D., R.Y.Y.T., J.T., M.T., H.S.M.); 2 Department of Radiology, Cambridge University Hospital NHS Foundation Trust, United Kingdom (T.D.).

**Keywords:** brain, leukoencephalopathy, magnetic resonance imaging, migraine with aura, encephalopathy

## Abstract

**Background and Purpose—:**

Cerebral autosomal dominant arteriopathy with subcortical infarcts and leukoencephalopathy (CADASIL) is the most common monogenic form of stroke usually presenting with migraine with aura, lacunar infarcts, and cognitive impairment. Acute encephalopathy is a less recognized presentation of the disease.

**Methods—:**

Data collected prospectively from 340 consecutively recruited symptomatic patients with diagnosis of CADASIL seen in a British National CADASIL clinic was retrospectively reviewed and original clinical records and imaging obtained. An encephalopathic event was defined as an acute event of an altered state of consciousness in a patient with CADASIL, manifesting with signs of brain dysfunction, which warranted hospital admission in the absence of any other cause. Clinical characteristics, risk factors, and outcome of encephalopathic presentations were studied.

**Results—:**

A total of 35 of 340 (10.3%) participants had a history of 50 encephalopathic events which was the first hospital presentation of CADASIL in 33 (94.3%) patients. Most commonly reported features during episodes were visual hallucinations (44%), seizures (22%), and focal neurological deficits (60%).Complete recovery within 3 months was reported in 48(96%) episodes. In 62% of episodes, there was a history of migraine or migraine aura directly preceding the encephalopathy. In 2 out of 15 cases where magnetic resonance imaging during episodes was available, unilateral focal cortical swelling was seen. A past history of migraine was independently associated with encephalopathy (odds ratio=12.3 [95% CI, 1.6–93.7]; *P*=0.015).

**Conclusions—:**

In up to 10% of CADASIL patients, a reversible encephalopathy is the first presentation leading to diagnosis. The strong association with migraine suggests a shared pathogenesis. Focal cortical swelling may be seen on magnetic resonance imaging during the acute episode.

## Abstract 2

Supplemental Digital Content is available in the text.

Cerebral autosomal dominant arteriopathy with subcortical infarcts and

leukoencephalopathy (CADASIL) is the most common form of familial stroke. It results from a small vessel arteriopathy because of mutations in the *NOTCH3* gene.^1^ Typical features include migraine usually with aura, lacunar strokes, depression, and later in life vascular cognitive impairment and dementia.

A less common, but increasingly recognized clinical manifestation is acute encephalopathy,^2,3^ also referred to as CADASIL coma, which has been reported to be the first major presenting feature in some CADASIL patients.^2^ This presentation is not always clinically recognized with many patients having been misdiagnosed as having infective encephalitis and treated accordingly.

It has been suggested that encephalopathy may be related to migraine,^4^ but the clinical features, predisposing factors, and prognosis remain poorly described. Furthermore, the pathogenesis is still unknown, and whether the episodes result in any permanent sequelae is uncertain.

We describe the clinical features, risk factors, imaging appearances, and outcome of CADASIL encephalopathy in a large prospectively collected cohort of patients with CADASIL.

We defined an encephalopathic event as an acute event of altered state of consciousness in a patient with CADASIL, manifesting with signs of brain dysfunction, which warrants hospital admission in the absence of any other cause.

## Methods

Data supporting the findings of this study is available from the corresponding author on reasonable request.

Data on 340 CADASIL patients seen in British CADASIL National Referral Centre was collected over a 20-year period between 1996 and 2016.The referral center sees patients from all over Great Britain with a confirmed or suspected diagnosis of CADASIL.

All patients in the study had a confirmed diagnosis of CADASIL, with a recognized pathogenic *NOTCH3* mutation, resulting in a change in a cysteine amino acid in one of the epidermal growth factor repeat (EGFr) domain in the extracellular portion of the NOTCH3 protein. We included only patients who had experienced at least one symptom of the disease, and asymptomatic individuals in whom CADASIL was detected on predictive testing were not included.

All patients were assessed by a consultant neurologist, and data were collected using a standardized questionnaire at first visit and follow-up encounters. The proforma included questions on the presence and age of onset of all known manifestations of CADASIL, including encephalopathic events, as well as signs, and symptoms experienced during those events.

A previous report described 6 cases of encephalopathy in the first 70 patients from the same cohort.^2^

## Standard Protocol Approvals, Registrations, and Patient Consents

Data were collected with local Ethics Committee approval (REC 16/EE/0118), and all patients gave written informed consent.

For the purpose of our study, the data from the prospectively collected database was then retrospectively reviewed, including demographics, data on cardiovascular risk factors and the type of *NOTCH3* mutations.

In addition to data collected on the proforma, hospital records, and magnetic resonance imaging (MRI) scans performed during encephalopathic episodes were reviewed.

A migraine was defined according to the International Classification of Headache Disorders

(ICHD). The first edition (ICHD-1) was used from 1996 to 2004, followed by ICHD-2 from

2004 to 2013 and ICHD-3 (β version) from 2013 to 2016.^5^ Stroke was defined clinically as a focal neurological episode lasting ≥24 hours because of a presumed vascular cause.

MRI scans performed during admission for encephalopathy were reviewed by a consultant neuroradiologist. We evaluated T1- and T2-weighted and diffusion-weighted images (DWIs). We also compared imaging abnormalities with those on follow-up or prevent MRI.

### Statistical Analysis

We compared clinical features and risk factors between CADASIL patients who had and had not experienced encephalopathy using 2 sample *t* test and Fisher exact tests as appropriate. Multiple logistic regression was then used to determine whether encephalopathy was independently associated with any variables, using the presence of encephalopathy as the outcome and history of stroke, migraine, age, and sex as variables.

To determine whether specific NOTCH3 mutation sites were associated with encephalopathy, mutations were mapped on the NOTCH3 protein using C-Bioportal Mapper (http://www.cbioportal.org/mutation_mapper.jsp).

Mutations were named according to human genome variation society nomenclature.^6^ We used the χ^2^ test to compare the frequency of mutation loci in the 2 groups, and the mutation position along the gene according to which of the 34 EGFr domains they are located in. Additionally, mutation location was grouped into EGFr 1 to 6 and EGFr 7 to 34, and the χ^2^ test was used to compare the groups.

Statistical analyses were performed using the R statistical software (version 3.2.2). Probability values of *P*<0.05 were considered statistically significant.

## Results

**Figure 1.**
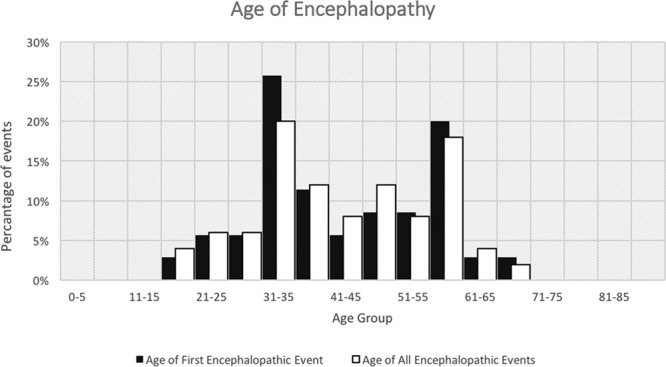
Histogram shows the percentage of patients in each age group during the first and all encephalopathic events.

Out of 340 CADASIL patients, 35 (10.3%) had experienced at least one encephalopathic event. In total, there were 50 events with 13 patients experiencing >1 event. The average age of patients at the time of their first encephalopathic event was 42 years, ranging between 19 and 62 (SD=12.9; Figure 1). In 33 (94%) of all encephalopathy cases, it was the first major symptom that leads to hospital admission or resulted in specialist neurological referral which led to the investigations and diagnosis of CADASIL. Many of these patients had previously experienced migraine, but this had not led to a diagnosis of CADASIL. In 2 (5.7%) affected cases encephalopathy was the first ever symptom of the disease. The most common diagnosis communicated to patients was meningitis or encephalitis, reported in 20 (40 %) of episodes. We know of 15 cases treated with antimicrobials. In addition, 2 cases were diagnosed as cerebral vasculitis of whom one was treated with steroids and azathioprine and one with steroids alone, with no improvement in symptoms noted. We were able to obtain lumbar puncture results for 14 episodes, although this is likely to be an underestimate of total number of lumbar punctures performed. In one case there was an isolated increase in cell count showing 11 cells/µl pleomorphic cells, with normal values of protein and glucose. In all the remaining cases the results were reported as normal. We were able to obtain EEG reports recorded during 5 encephalopathic episodes. This included 2 with clinical seizures during the course of admission, which showed isolated abnormalities over left anterior temporal area in one case and slow activity in the left hemisphere in the other. In addition, 3 episodes without seizures had EEGs; of these one had abnormal right temporal background activity, one bilateral slow waves especially in the left temporal lobe, and one had no specific changes.

### Features of the Events

Features of the events and availability of the investigations are presented in Table I in the online-only Data Supplement.

Typically the encephalopathic episode began with migrainous symptoms progressing without any break to a period of reduced consciousness during which patient would develop additional neurological signs.

A migraine or isolated migrainous aura immediately preceded 31 (62.0%) of the 50 episodes. In addition, some patients experienced nonmigraine type headache later during the episodes and, therefore, any type of headache was a feature in 36 (72.0%) of episodes.

Seizures were reported in 11 events (22.0%). These were described as generalized or tonic-clonic events (7 episodes), and complex partial seizures (2 episodes), unspecified seizure type (2 episodes). None of these patients suffered from seizures before the episode. In one case seizures recurred about 8 months following the encephalopathic presentation.

Focal neurological signs during the events included unilateral weakness (9 episodes 18.0%), unilateral sensory deficit (7 episodes 14.0%), dysarthria (6 episodes 12.0%), aphasia (11 episodes 22.0%), neglect or inattention (4 episodes 8.0%), apraxia (2 episodes 4.0%), visual agnosia (2 episodes 4.0%), and dyslexia (2 episodes 4.0%). In total, 30 (60%) events where accompanied by at least one of the above.

Out of the total 50 episodes, 22 (44.0%) were accompanied by visual hallucinations. Reports of hallucinations included absence of lower half of people’s body (metamorphopsia; 1 case), people, dead people or ghosts (5 cases), and animals (2 cases) as well as more complex hallucinations such as blood pressure machine turning to evil spirit or boyfriend on the ward with another woman. Paranoid delusions were reported in 2 (4.0%) episodes. As it was a criterion for diagnosis of an encephalopathic event, reduced consciousness was a feature in all patients ranging between confusion and coma.

The duration of longest hospital admission was available for 25 (71.4%) patients. It varied between 1 and 21 days with a mean duration of 9.7 days (SD=5.5). Encephalopathic episodes recurred in 13 of 35 subjects (37.1%) with 1, 2, 3, and 4 recurrent events occurring in 6, 3, and 4 individuals, respectively.

Complete patients’ recovery within 1 week was noted for 12 (24%) episodes, within 2 weeks for 20 (40%), within 1 month 37 (74 %), and within 3 months for 48 (96%).

Reported residual deficits after discharge included short-lived (3 weeks) unilateral sensory disturbance (1 case) and cognitive decline (4 cases). Cognitive assessment, 1 week postevent for one case, revealed an impairment of spatial and visuoperceptual processing as well as executive dysfunction with normal verbal and abstraction skills (Mini-Mental State Examination of 27/30).This was followed by gradual recovery to baseline function. In the second case assessment about 2 weeks after the event showed reduction in visual processing speed and working memory with normal verbal fluency (Mini-Mental State Examination 26/30) again followed by gradual recovery to baseline. In 2 other cases, nonspecific worsening of cognitive symptoms was reported following the event with no subsequent recovery noted.

### Risk Factors

Demographics, clinical, and cardiovascular risk factors were compared between CADASIL patients with and without a history of encephalopathy and are shown in the Table. The only factors that differed significantly on simple logistic regression were migraine as a first symptom, and history of migraine, which were more common in the encephalopathy group, and history of stroke, which was less common in the encephalopathy group.

**Table.**
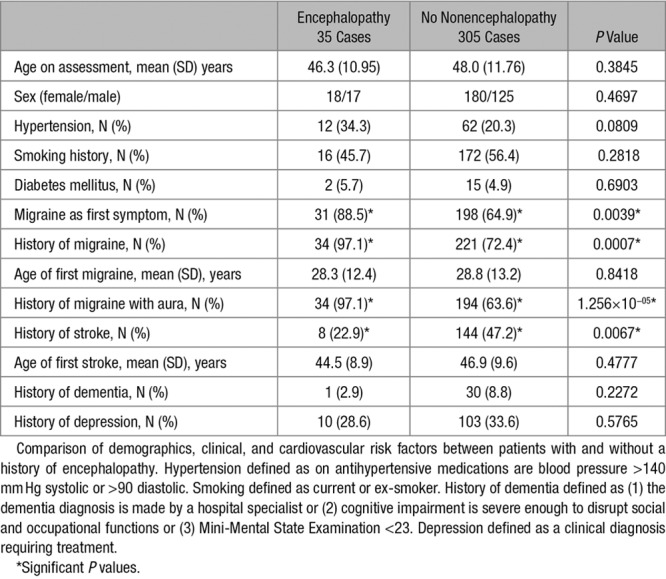
Characteristics of Patients Who Experienced Encephalopathy Compared With the Rest of the Cohort

To determine which risk factors were independent predictors of encephalopathy, multiple logistic regression was performed entering covariates found to be significantly associated with encephalopathy on simple logistic regression (history of migraine and history of stroke) as well as age and sex. On multiple logistic regression, history of migraine was independently associated with a history of an encephalopathic event (odds ratio, 12.3 [95% CI, 1.6–93.7]; *P*=0.015) and history of stroke was negatively associated with a history of encephalopathy (odds ratio, −0.41 [95% CI, −0.96 to −0.17]; *P*=0.04).

### Relation to Genotype

**Figure 2.**
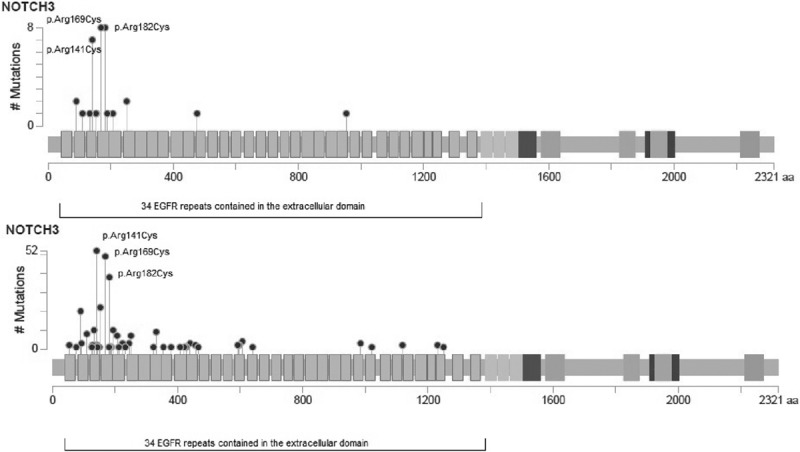
Distribution of mutations in the NOTCH3 protein among patients with history of encephalopathy (**top**) and without history of encephalopathy (**bottom**). *x* axis: amino acids along the protein; including 34 epidermal growth factor repeat (EGFr) domains in the extracellular domain(marked); *y* axis: number of cases where each lollipop represents a different mutation site. Three most frequent mutations marked. Image created with C-bioportal. Mapper. (http://www.cbioportal.org/mutation_mapper.jsp).

The location of the NOTCH3 mutation was available for all patients. The distribution of mutations is shown in Figure 2. There was no apparent difference in the distribution between cases with and without encephalopathy.

There was no difference in the EGF repeat in which the mutation occurred between cases with and without encephalopathy (χ^2^, *P*=0.388) and in the proportion who had a mutation in EGFR repeat 1 to 6 versus 7 to 34 (encephalopathy 33 v 2 versus nonencephalopathy 256 v 49, χ^2^ *P*=0.170).

The most common mutation sites were the same in both groups (p.Arg182Cys, p.Arg169Cys, and

p.Arg141Cys) and there were no differences in the frequency of these mutations between both groups (p.Arg182Cys, *P* value=0.11; p.Arg169Cys, *P* value=0.34; and p.Arg141Cys, *P* value=0.82).

## Imaging

Brain MRI scans were available in all cases but MRIs performed during an encephalopathic episode were available for review for 15 episodes. T1, T2-weighted, and FLAIR images were obtained for all 15 episodes and DWIs for 8 episodes. All scans were performed using 1.5 Tesla scanners in neurology or medical units of the local hospitals. Mean (SD) time between admission and MRI was 6.3 (SD=7.4) days, with time ranging between within 24 hours and 18 days.

**Figure 3.**
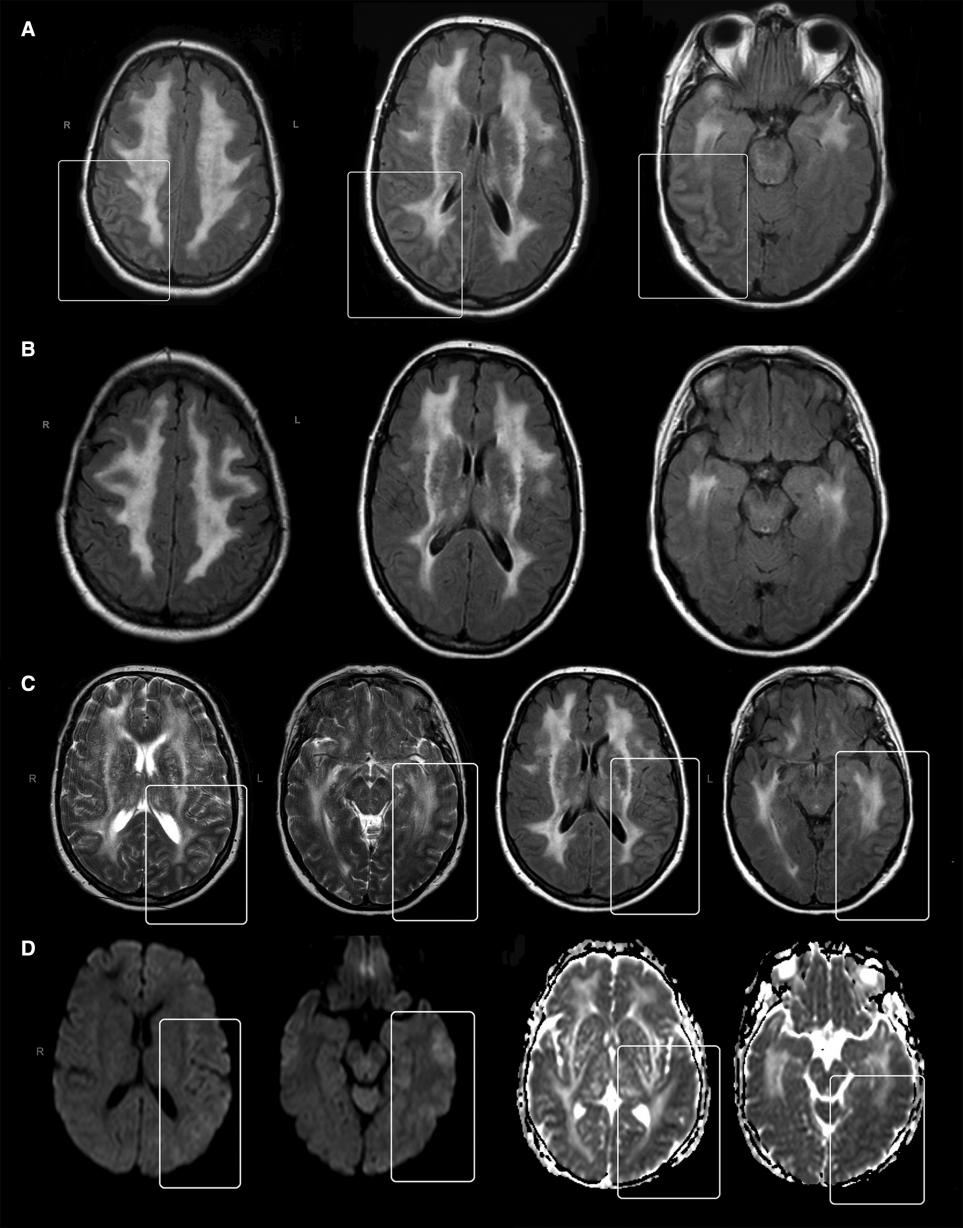
Patient 1 during first episode. Flair images revealing increased signal and swelling in the right temporal and parietal regions (approximate area of changes marked) (**A**) with near complete resolution of changes on follow-up images 40 days later (**B**). Patient 1 during the second episode. T2 weighted and flair images (**C**) revealing cortical swelling and signal abnormalities in the left parietal and temporal region (marked area). Diffusion-weighted image (DWI) images are showing mild increase in signal with corresponding low signal on apparent diffusion coefficient map (**D**).

All 15 cases had MRI showing signs of moderate to severe cerebral small vessel disease. There were no focal regions of impaired diffusion consistent with acute ischemia in any of the 8 cases with DWI available. In 2 patients, MRI revealed unilateral focally increased signal intensity with swelling and increased volume of the involved cortical gyri on T2-weighted images. In the first case, the lesions were located in the cortical gray matter and subcortical white matter of the right parietal, temporal and occipital gyri and during the second event affecting the same patient, 4 years later, in the left parietal, and left medial and lateral temporal gyri extending to left occipital lobe (Figure 3). DWI during the second event revealed subtle increased signal intensity in the peripheral cortical gray matter with corresponding hypodensity on apparent diffusion coefficient map. Follow-up images available 40 days after the first event revealed near complete resolution of the findings (Figure 3). In the second patient, similar lesions were present in the left parietal, left lateral temporal, and occipital areas. The second patient had a generalized seizure during the event. No seizure-like activity was reported in the first case.

## Discussion

In this large cohort of CADASIL patients, encephalopathy occurred in ≈10% of individuals. Importantly, it was the first major symptom leading to specialized neurological or hospital presentation in over 90% of individuals with encephalopathy. In many cases, the diagnosis was delayed and alternative treatment given, particularly for infective encephalitis, which emphasizes the importance of awareness of this syndrome.

This study highlights the typical features of CADASIL encephalopathy. In contrast to the subcortical features of ischemic damage occurring in CADASIL with lacunar stroke syndromes, CADASIL encephalopathy was associated with a range of cortical symptoms and signs, including aphasia, neglect and inattention, apraxia, visual agnosia. Although a rare feature of CADASIL outside of encephalopathic episodes, seizures were frequent during encephalopathic episodes and tended not to recur outside the acute illness. Visual hallucinations were also a prominent feature occurring in almost 50% of encephalopathic episodes and paranoid delusions were occasionally reported.

Encephalopathic episodes tended to be self-terminating and patients recovered within days to a few weeks without specific treatment. They did recur in more than one-third of the patients with 5 being the maximum number of episodes in an individual patient. Episodes were usually accompanied by full recovery (96% of cases) although there were rare reports of persisting cognitive symptoms; however, detailed long-term assessment in these cases was not available.

The major risk factor for encephalopathy was migraine; we found a strong association between a past history of migraine and risk of encephalopathy. Furthermore, in over 60% of encephalopathic episodes, there was a history of migraine immediately preceding the episode. This proportion is likely to be underestimated as some patients had poor recollection of the initial symptoms before the encephalopathy. This high prevalence of migraine as preceding symptom is consistent with previously reported case series.^2,3^

In some cases, drawing a distinction between confusional migraine and encephalopathic episodes was difficult. Although unclassified by the ICHD-III-2013^5^ confusional migraine is also well described in sporadic migraine.^7^ We defined an encephalopathic event as an acute event of an altered state of consciousness in a patient with CADASIL, manifesting with signs of brain dysfunction, which warranted hospital admission in the absence of any other cause. We understand that hospital admission might seem an arbitrary inclusion considering that confusional migraine is not uncommon in CADASIL.^8^

Rare cases of migraine in patients without CADASIL have been associated with coma.^9,10^ Holub et al^11^ described a fatal case of migraine in a 12-year-old girl with a history of repeated loss of consciousness. Furthermore, a similar presentation referred to as coma or encephalopathy has been reported in several cases of familial hemiplegic migraine, another genetic form of migraine.^12,13^

Apart from the association with migraine, the only other association we found was a weakly significant inverse association between stroke and encephalopathy. This reflects a similar inverse relationship between the history of migraine and stroke reported in the same cohort^4^ but needs replication in further populations.

There is little previous information available on brain imaging appearances during CADASIL encephalopathic episodes. DWI images in our study were available for the majority of patients but these were usually performed during the nonacute phase. DWI scans during the acute encephalopathic episode were only available for 8 cases.

In those patients who had DWI, there were no instances of acute ischemic lesions. In 2 patients, we found changes of cortical swelling and hyperintensity on T2-weighted imaging consistent with cerebral edema. Such findings have been reported in status migrainosus and in both sporadic and familial hemiplegic migraine.^14,15^ Cerebral edema can also occur secondary to status epilepticus but only one of the 2 patients had seizures and these were not prolonged. Despite no overt signs of acute ischemia, in one of those cases, there were subtle diffusion changes suggesting presence of mild cytotoxic edema. Similar changes have been previously observed in cases of familial hemiplegic migraine.^16^

The cause of encephalopathy in CADASIL remains uncertain. The close association with migraine, and the evolution of encephalopathic episodes from migraine, suggests shared pathophysiological mechanisms.

Cortical spreading depression (CSD) is characterized by appearance of depolarization waves spreading at a slow velocity across the cortical surface which is a complex event that coincides with, and is caused by, a dramatic failure of brain ion homeostasis and efflux of excitatory amino acids from nerve cells.^17^ Reports on symptoms of migrainous aura explained by excitation-depression wave propagating across the human primary visual cortex at the same rate as CSD suggest a possible link between the 2.^18^ Imaging of cerebral blood flow in patients during migraine attacks has shown reduced blood flow that propagated across the cortex at the same rate and with similar signs of vascular impairment as CSD.^19^ These studies provide indirect evidence to support the view that CSD could be an underlying mechanism for migrainous aura. Consistent with a possible role for CSD in migraine increased susceptibility to CSD has been shown in transgenic NOTCH3 mice.^20^ The strong relation between encephalopathy and migraine with aura that we have shown might be the result of a shared pathogenesis. The position of the mutation along the *NOTCH3* gene has suggested as a determinant of the spectrum and severity of CADASIL disease,^21^ but we found no association between the location of the mutation in the *NOTCH3* gene and encephalopathy risk. It is also possible that other genetic factors may influence risk, and it has been shown that modulating genes seem to influence the severity of white matter damage in patients with CADASIL^22^ although the responsible genes have not been identified.

Our study does have some limitations. Although using a prospectively collected database, it was a retrospective study and in many cases, we were not able to obtain MRI images or cerebrospinal fluid results performed during admission. We do not have reliable data on consciousness level as events happened in multiple hospitals and we did not have access to nursing records or observation charts which would include this.

In many cases, we could not establish whether the focal neurological signs were part of an initial migraine with aura or developed later in the course of encephalopathic episode. Similarly, there is a clinical overlap between confusional migraine and encephalopathic episodes described in CADASIL patients, and those 2 might represent different ends of the same spectrum.

## Conclusions

Our results emphasize that encephalopathy is an important feature of CADASIL and may be the first major presenting symptom, and therefore, requires a high index of suspicion. The diagnosis of CADASIL encephalopathy should be considered in patients with a known diagnosis of CADASIL, or typical white matter changes present on neuroimaging, who present with a prolonged episode of confusion or loss of consciousness, and who may also have seizures of cortical signs. Although its mechanism remains uncertain encephalopathy is closely associated with migraine with aura, and an increased propensity to spreading depression may play a role in the increased frequency of both migraine with aura and encephalopathy seen in CADASIL.

## Sources of Funding

Drs Drazyk and Traylor are funded by a British Heart Foundation programme grant (RG/16/4/32218). H.S. Markus is supported by a National Institute for Health Research Senior Investigator award. His work, and that of the Stroke Research Group, is supported by the Cambridge University Hospital National Institute for Health Research Biomedical Research Centre. J. Tay is funded by a PhD studentship from Cambridge Trust. Dr Tan is supported by the Agency for Science, Technology and Research Singapore (MBBS-PhD scholarship). The funders had no role in study design, data collection and analysis, decision to publish, or preparation of the article.

## Disclosures

Dr Drazyk participated in the study concept and design, acquisition of data, analysis and interpretation of data, and wrote the first draft article. Dr Tan participated in the acquisition of data and critical revision of the article for intellectual content. J. Tay participated in the statistical analysis and critical revision of the article for intellectual content. Dr Traylor participated in the statistical analysis of genetics data and critical revision of the article for intellectual content. Dr Tilak participated in the analysis of imaging data and critical revision of the article for intellectual content. H. Markus participated in the study concept and design, acquisition and interpretation of data, critical revision of the article for intellectual content, and study supervision.

## Supplementary Material
