# Supplementary material for: Encephalopathy in a Large Cohort of British Cerebral Autosomal Dominant Arteriopathy With Subcortical Infarcts and Leukoencephalopathy Patients
Source: Stroke. 2019 Jan 14;50(2):283–90. doi: 10.1161/STROKEAHA.118.023661 (PMC6358181; doi:10.1161/STROKEAHA.118.023661)
Supplement: Supplementary file 1 [file str-50-283-s001.pdf]

Encephalopathic Events	50(35 patients)	A	B	C	D	D1	E	F	G	H	I	J	K	L	M	N	O	P	P1	Q	R	S	T	U
Age of First Encephalopathic Event	**mean(42,7)**	23	42	66	58		30	34	59	29	46	46	33	53	52	35	34	33		31	38	51	25	44
First feature of CADASIL **N(%)**	**2(5.71)**															✓							✓	
First feature requiring admission **N(%)**	**33(94,2)**	✓	✓	✓	✓		✓	✓	✓	✓	✓	✓	✓	✓	✓	✓	✓	✓		✓	✓	✓	✓	✓
**Clinical features n(%)**																								
Preceeded by migrainous headache/aura	**31(62)**	✓	✓		✓	✓	✓		✓	✓	✓		✓		✓		✓	✓	✓	✓		✓		
Headache	**36(72)**	✓	✓	✓	✓	✓	✓		✓	✓	✓	✓	✓		✓	✓	✓	✓	✓	✓		✓	✓	✓
Seizure	**11(22)**			✓						✓							✓	✓			✓			
Hallucinations	**22(44)**		✓		✓	✓	✓				✓						✓							
Aphasia	**11(22)**	✓		✓										✓			✓	✓	✓			✓		
Hemiparesis	**13(26)**	✓	✓							✓	✓			✓			✓			✓				
Hemisensory	**7(14)**										✓				✓		✓							
Dysarthria	**6(12)**											✓		✓						✓				
Dyslexia	**2(4)**																	✓						
Neglect/Inattention	**4(8)**			✓				✓									✓							✓
Apraxia	**2(4)**							✓																✓
Visual perception deficit	**2(4)**							✓																
Paranoid delusions	**2(4)**							✓																
Complete Recovery within 1 week	**12(24)**				✓	✓									✓							✓	✓	
Complete Recovery within 2 week	**20(40)**				✓	✓			✓		✓				✓		✓					✓	✓	
Recovery within 1 month	**37(74)**	✓			✓	✓		✓	✓	✓	✓	✓		✓	✓		✓			✓		✓	✓	
Complete Recovery within 3 months	**48(76)**	✓	✓	✓	✓	✓	✓	✓	✓	✓	✓	✓	✓	✓	✓	✓	✓	✓	✓	✓	✓	✓	✓	
Patients who experienced recurrence **N(%)**	**11 (22)**				✓													✓						
MRI available	**15(30)**		✓	✓			✓	✓				✓	✓					✓			✓			
CSF result available	**14(28)**								✓	✓	✓				✓		✓	✓					✓	

Encephalopathic Events	W	W1	X	X1	Y	Z	Z1	Z2	AA	AA1	BB	CC	CC1	DD	DD1	FF	FF1	FF2	GG	GG1	HH	HH1	HH2	HH3	II	JJ	KK
Age of First Encephalopathic Event	63		38		58	34			32		59	59		30		37			19		47				57	60	39
First feature of CADASIL **N(%)**																											
First feature requiring admission **N(%)**	✓				✓	✓			✓		✓			✓		✓			✓		✓				✓	✓	✓
**Clinical features n(%)**																											
Preceeded by migrainous headache/aura	✓	✓	✓	✓	✓	✓	✓	✓	✓	✓				✓	✓			✓	✓	✓					✓		
Headache	✓	✓	✓	✓	✓	✓	✓	✓	✓	✓				✓	✓			✓	✓	✓					✓		
Seizure						✓	✓		✓		✓							✓	✓								
Hallucinations		✓	✓		✓	✓	✓		✓	✓	✓			✓	✓	✓	✓	✓				✓			✓		✓
Aphasia	✓				✓											✓	✓										
Hemiparesis				✓		✓	✓			✓								✓		✓							
Hemisensory							✓													✓			✓				
Dysarthria												✓	✓											✓			
Dyslexia																✓											
Neglect/Inattention																											
Apraxia																											
Visual perception deficit					✓																						
Paranoid delusions										✓																	
Complete Recovery within 1 week			✓	✓						✓			✓	✓	✓		✓										
Complete Recovery within 2 week			✓	✓						✓	✓	✓	✓	✓	✓		✓					✓			✓	✓	
Recovery within 1 month	✓		✓	✓	✓	✓		✓	✓	✓	✓	✓	✓	✓	✓		✓		✓	✓	✓	✓	✓	✓	✓	✓	✓
Complete Recovery within 3 months	✓		✓	✓	✓	✓	✓	✓	✓	✓	✓	✓	✓	✓	✓	✓	✓	✓	✓	✓	✓	✓	✓	✓	✓	✓	✓
Patients who experienced recurrence **N(%)**	✓		✓			✓			✓			✓		✓		✓			✓		✓						
MRI available	✓					✓					✓			✓		✓									✓		✓
CSF result available	✓				✓						✓								✓		✓					✓	✓

SUPPLEMENTAL MATERIAL

Table I. Features of 50 encephalopathic events in 35 CADASIL patients including availability of investigations. n- number of events, N –number of patients.
